# Can white matter hyperintensities based Fazekas visual assessment scales inform about Alzheimer’s disease pathology in the population?

**DOI:** 10.21203/rs.3.rs-4017874/v1

**Published:** 2024-03-11

**Authors:** Aishwarya Pradeep, Sheelakumari Raghavan, Scott A. Przybelski, Gregory Preboske, Christopher G. Schwarz, Val J. Lowe, David S. Knopman, Ronald C. Petersen, Clifford R. Jack, Jonathan Graff-Radford, Petrice M. Cogswell, Prashanthi Vemuri

**Affiliations:** Mayo Clinic Alix School of Medicine; Mayo Clinic; Mayo Clinic; Mayo Clinic; Mayo Clinic; Mayo Clinic; Mayo Clinic; Mayo Clinic; Mayo Clinic; Mayo Clinic; Mayo Clinic; Mayo Clinic

**Keywords:** white matter hyperintensities, Alzheimer’s disease, Fazekas score, periventricular white matter hyperintensity, deep white matter hyperintensity

## Abstract

**Background:**

White matter hyperintensities (WMH) are considered hallmark features of cerebral small vessel disease and have recently been linked to Alzheimer’s disease pathology. Their distinct spatial distributions, namely periventricular versus deep WMH, may differ by underlying age-related and pathobiological processes contributing to cognitive decline. We aimed to identify the spatial patterns of WMH using the 4-scale Fazekas visual assessment and explore their differential association with age, vascular health, Alzheimer’s imaging markers, namely amyloid and tau burden, and cognition. Because our study consisted of scans from GE and Siemens scanners with different resolutions, we also investigated inter-scanner reproducibility and combinability of WMH measurements on imaging.

**Methods:**

We identified 1144 participants from the Mayo Clinic Study of Aging consisting of older adults from Olmsted County, Minnesota with available structural magnetic resonance imaging (MRI), amyloid, and tau positron emission tomography (PET). WMH distribution patterns were assessed on FLAIR-MRI, both 2D axial and 3D, using Fazekas ratings of periventricular and deep WMH severity. We compared the association of periventricular and deep WMH scales with vascular risk factors, amyloid-PET and tau-PET standardized uptake value ratio, WMH volume, and cognition using Pearson partial correlation after adjusting for age. We also evaluated vendor compatibility and reproducibility of the Fazekas scales using intraclass correlations (ICC).

**Results:**

Periventricular and deep WMH measurements showed similar correlations with age, cardiometabolic conditions score (vascular risk), and cognition, (p < 0.001). Both periventricular WMH and deep WMH showed weak associations with amyloidosis (R = 0.07, p = < 0.001), and none with tau burden. We found substantial agreement between data from the two scanners for Fazekas measurements (ICC = 0.78). The automated WMH volume had high discriminating power for identifying participants with Fazekas ≥ 2 (area under curve = 0.97).

**Conclusion:**

Our study investigates risk factors underlying WMH spatial patterns and their impact on global cognition, with no discernible differences between periventricular and deep WMH. We observed minimal impact of amyloidosis on WMH severity. These findings, coupled with enhanced inter-scanner reproducibility of WMH data, suggest the combinability of inter-scanner data assessed by harmonized protocols in the context of vascular contributions to cognitive impairment and dementia biomarker research.

## BACKGROUND

White matter hyperintensities (WMH) of vascular origin are hyperattenuating lesions detected on T2-weighted or fluid-attenuated inversion recovery (FLAIR) brain magnetic resonance imaging (MRI) sequences. WMH have increasingly been recognized as a manifestation and marker of cerebral small vessel disease (SVD) [1, 2] and are due to multiple mechanisms such as hypoperfusion or blood brain dysfunction which ultimately leads to cell death and myelin loss [3]. WMH increases and expands in volume in individuals with hypertension [4–6] and with age [6, 7], and are associated with increased risk of stroke, dementia, and mortality [9]. There is considerable heterogeneity in the patterns of WMH burden, ranging from small punctate lesions measuring < 10 mm, to single and/or multiple areas of emerging confluence measuring < 20 mm, and large confluent areas measuring > 20 mm. The most common classification of WMHs is by location as periventricular white matter hyperintensities (PWMH) which develop proximal to the lateral ventricles and deep white matter hyperintensities (DWMH) in the subcortical white matter regions [10]. Furthermore, PWMH and DWMH can be distinguished by their underlying pathophysiological mechanisms. While PWMH is believed to arise from endothelial destruction and interstitial edema near the ventricles [11–13], DWMH originates from demyelination, gliosis, and axonal cleavage [13–15]. As a result, ischemic pathology may directly affect myelination, whereas veno-occlusive disease may block interstitial fluid resorption and cause edema [13]. Numerous methods exist to measure the extent of WMH burden, both semi-qualitative visual rating scales such as Fazekas [16], Manolio [17], and Scheltens [18], and quantitative volumetric methods of measurement that are manual, semi-automated, or fully automated [10]. Our project utilized the Fazekas scale due to its advantage in evaluating PWMH and DWMH burden separately. Furthermore, they are increasingly used for assessment of vascular contributions to cognitive impairment and dementia (VCID) based on the Fazekas score of 2 or higher for PWMH and/or DWMH along with symptoms of cognitive impairment.

While the strong correlation between WMH burden and SVD is well-established, there is growing interest in exploring the potential link between WMH and neurodegenerative diseases (which frequently co-occurs with SVD), such as Alzheimer’s disease (AD), the most prevalent cause of age-related dementia in the general population [19]. There is evidence that both amyloid and tau pathologies seen in AD are associated with changes in WMH. McAleese et al. found an independent and strong correlation between high levels of cortical tau load and WMH burden [20]. In addition, high burden of WMH has been seen in autosomal dominant AD and early onset AD studies where the risk of SVD is low due to the younger age. Given the recent data on the links between WMH burden and AD severity and use of Fazekas visual scales for assessment of VCID, there is an unmet need to investigate if the Fazekas visual scales are reflective of AD pathological burden, specifically PWMH, based on the location of AD related changes seen in WMH.

The primary goal of this study was to evaluate whether WMH subtypes ranked based on Fazekas visual scales for PWMH and DWMH exhibit differential associations with AD and/or SVD. We hypothesized that PWMH grading will be associated with AD pathology. This investigation allowed us to evaluate the potential mechanistic links between spatial heterogeneity in WMH and progression of AD. As a secondary goal, we wanted to determine the combinability of 2D-FLAIR (which has historically been used for assessment of WMH and has excellent in-plane signal-to-noise ratio) and 3D-FLAIR (which are increasingly being used recently for quantitative assessment of WMH and has higher 3D spatial resolution) based on Fazekas scores as well as cross-vendor compatibility between GE 2D FLAIR and Siemens 3D FLAIR scanners using the specified adjustments in the algorithm that allow for it.

## METHODS

### Selection of participants

Participants for this study were enrolled through the Mayo Clinic Study of Aging (MCSA), a population-based study of older adult residents in Olmsted County, Minnesota, with entry age ranging from 30 to 89 [21, 22]. The design and diagnostic/selection criteria for participants of this study has been previously published. Data for the MCSA was enumerated from the Rochester Epidemiology Project (REP) medical records linkage system. Electronic medical records were reviewed for all patients and pertinent information on vascular health was obtained in depth. We included 1144 participants who underwent 2D axial FLAIR-MRI (AFL) or 3D FLAIR-MRI (3FL), amyloid-PET, and tau-PET scans. The combinability study comparing AFL and 3FL had a mean age of 77 years (ranges from 58–97) and consisted of 76 individuals with both types of FLAIR scans.

### Standard protocols, approvals, registration, and patient consent

The study was approved by the Mayo Clinic and Olmsted Medical Center Institutional Review Board (IRB) and written informed consent was obtained from all participants or their surrogates.

### Vascular health indicator

We used the nurse-abstracted vascular risk data from electronic health records that included the history of seven conditions defined by the United States Department of Health and Human Services, which were summated to a composite score providing a comprehensive representation of an individual’s cardiometabolic condition (CMC): cardiac arrhythmias, diabetes mellitus, hyperlipidemia, hypertension, coronary artery disease, congestive heart failure, and stroke [23].

### Assessment of amyloid and tau-PET measures

Amyloid-PET imaging was conducted using PiB-PET and tau-PET with 18F-AV-1451. The detailed descriptions of acquisition, processing, and summary measure calculation for amyloid and tau-PET scans were published previously [24]. The global amyloid load, i.e., standardized uptake value ratio (SUVR) for each participant was calculated by attaining the median uptake value in the prefrontal, orbitofrontal, parietal, temporal, anterior cingulate, and posterior cingulate/precuneus regions of interest (ROIs) and dividing by the median uptake in the cerebellar crus grey matter ROI. Global tau SUVR for each participant was calculated by obtaining the median tau-PET uptake in the entorhinal, amygdala, parahippocampal, fusiform, inferior temporal, and middle temporal ROIs normalized by the median tau-PET uptake in the cerebellar crus grey matter ROI.

### Assessment of white matter hyperintensities

Images were acquired as 2D FLAIR-MRI scans with a 3T GE scanner (GE Medical Systems, Milwaukee, WI) and as 3D FLAIR-MRI scans with a 3T Siemens scanner (Siemens Medical Solutions, Malvern, PA). The 2D T2-weighted FLAIR- MRI scans were obtained with the following parameters: repetition time = 11 000 ms, echo time = 147 ms, inversion time = 2,250ms, 256 × 192 matrix, 24-cm field of view, and voxel size = 0.86 × 0.86 × 3 mm. 3D FLAIR images were acquired with the parameters as follows: TR = 5000 ms, TE = 388 ms, slice thickness = 1 mm, inversion time = 1800 ms, matrix size = 256 × 100, and voxel size = 1 × 1× 1 mm.

The complete details of acquisition of white matter hyperintensity segmentation procedures have been previously published and both 2D (GE) and 3D (Siemens) T2 FLAIR were segmented using a fully automated in-house algorithm [25]. This method internally detects scanner manufacturer and 2D vs. 3D FLAIR and the algorithm is adjusted to produce more consistent values across these favors. We also scaled the volume of WMH by total intracranial volume (WMH/TIV%) to account for head size.

Acquired images were rated by a medical student (A.P) blinded to all participant identifiers and medical record details and trained to utilize the Fazekas visual rating scale. We excluded participants who demonstrated the following on imaging: old or new ischemic or hemorrhagic infarcts in cortical areas or lesions in the brainstem, basal ganglia, cerebellum, thalamus, midbrain, pons, medulla. We implemented Fazekas as a semi-qualitative measure assessing location (periventricular and deep white matter) and degree of pathologic WMH spread by assigning a PWMH and DWMH rating to each participant. A scale of 0 to 3 was used for both PWMH and DWMH, with higher rating correlating with increased severity and spread, with 0 as none, 1 as mild, 2 as moderate, and 3 as severe.

**Figure 1:**
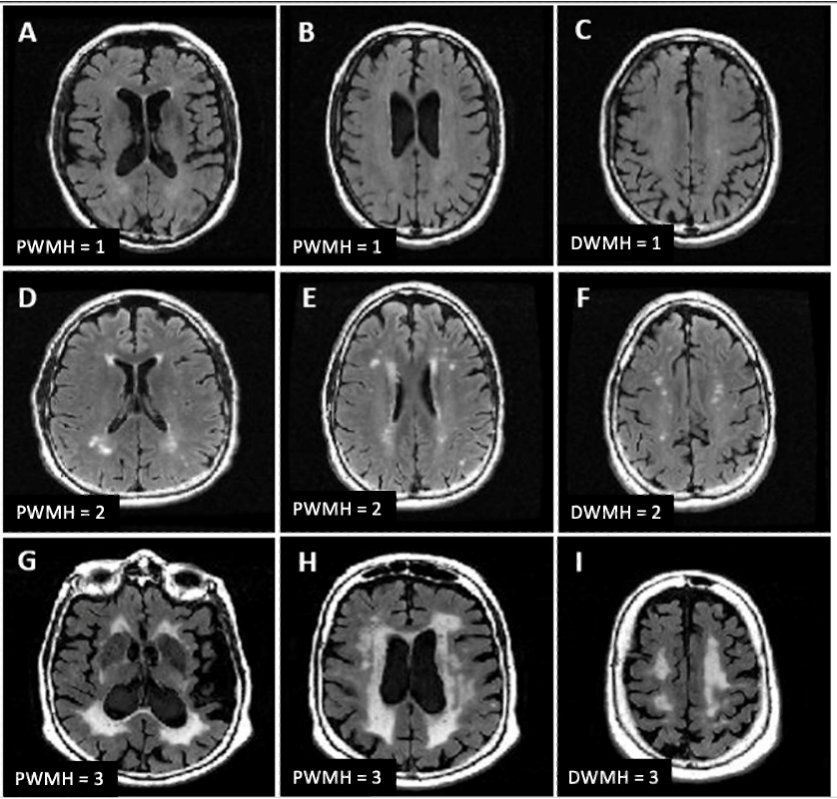
PWMH = 1: small caps **(A),** pencil-thin lining **(B)**; DWMH = 1: punctate deep white matter lesions **(C)**; PWMH = 2: large caps **(D)** smooth halo **(E**); DWMH = 2: beginning of confluence **(F)**; PWMH = 3: extending caps **(G),** irregular periventricular white matter lesions ; DWMH = 3: confluent deep white matter lesions **(I).**

For PWMH, grade 1 lesions were defined as pencil-thin lining or caps around the lateral ventricles, 2 were irregular hyperintensities forming a smooth halo, and 3 were lesions spreading from the ventricles into the surrounding deep white matter. For DWMH, grade 1 lesions indicated punctate shaped hyperintensities, 2 were punctate hyperintensities with tendency to fuse together (beginning of confluence), and 3 were large, fused punctate hyperintensities. All participants had some degree of PWMH, warranting a minimum rating of 1 for PWMH. The criteria used for a given PWMH and DWMH rating are outlined in Fig. 1.

### Cognitive measurements

Participants underwent the MCSA neuropsychological battery consisting of nine tests assessing four cognitive domains: (1) memory (Wechsler Memory Scale–Revised (WMS-R) Logical Memory-II (delayed recall), WMS-R Visual Reproduction-II (delayed recall), Auditory Learning Verbal Test delayed recall), (2) attention-executive function (Trail Making Test Part B and Wechsler Adult Intelligence Scale–Revised (WAIS-R) Digit Symbol), (3) language (Boston Naming Test and category fluency), and (4) visuospatial skills (WAIS-R Picture Completion and WAIS-R Block Design). The aggregate test performance results for each participant were tabulated as a global cognitive z-score that was estimated from the z-transformation of the average of the z-scores of the four domains weighted back to the Olmsted County population.

### Statistical Analysis

We report the means and standard deviations (SD) for continuous demographic and clinical variables along with the counts and percentages for categorical variables. Group differences were analyzed with one-way analysis of covariance (ANCOVA) for continuous and logistic regression for categorical variables where an adjustment for age was included. Amyloid-PET, tau-PET and WMH were analyzed with a log transformation due to skewness. We conducted visual Fazekas scale rating assessments on all GE (2D FLAIR) and Siemens (3D FLAIR) scans separately. Kendall Tau Partial Correlations were done comparing the Fazekas visual rating with CMC, cognition and imaging measures which were adjusted for age. We conducted sensitivity analyses with data from 74 cross-over participants (participants scanned on both scanners) with both assessments on 2D FLAIR and 3D FLAIR images scanned within the same week. For vendor compatibility comparison a combination of weighted Kappas, intraclass correlations, and Pearson correlations were performed. We utilized Youden’s J statistic to determine the optimal cut-off threshold which maximizes the distance to the identity line that provides the overall best sensitivity and specificity. Area under the receiver operation curves (AUROC) were calculated to distinguish the difference between those with abnormal Fazekas score from normal Fazekas score. We used p < 0.05 (two-tailed) to determine statistical significance in all analyses.

## RESULTS

The characteristics of the participants are summarized in Table 1. The participants scanned on GE and Siemens scanners are shown both separately and combined. Overall, the mean (standard deviation) age was 74.0 (8.8) years, 53% were male, 29% were APOe4 carriers, 42% were amyloid positive, and 30% were tau positive. Cognitively unimpaired individuals comprised 87% of this sample. Out of 1144 participants, 524 had 2D axial FLAIR-MRI (AFL) and 620 had 3D FLAIR-MRI (3FL). Four participants did not have a PiB, 3 did not complete tau-PET, and 8 did not have total WMH burden assessment due to quality control failures and missing data.

### Association of age, CMC, and Global z-score with PWMH and DWMH burden

**Figure 2:**
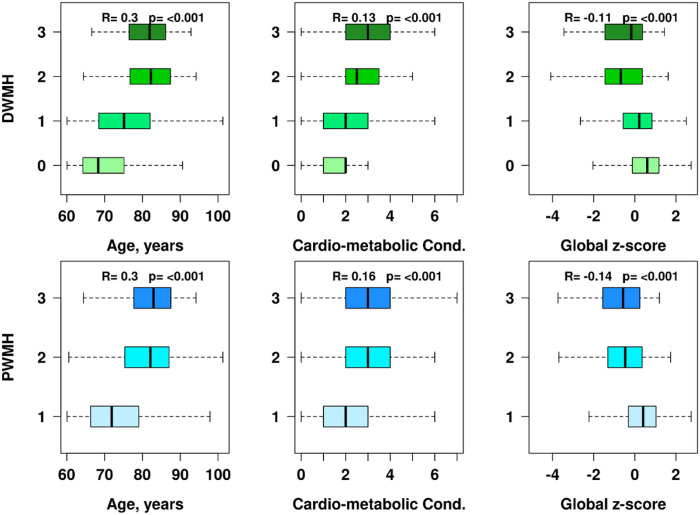
Box plots of age (years), cardiovascular and metabolic conditions (CMC), and cognitive function (Global z-score) as a function of DWMH (top panel) and PWMH (bottom panel) Fazekas grading scales. The Kendall tau correlations and p-values are listed.

The box plots in Fig. 2 report Kendall tau correlations and corresponding *p*-values. There is a positive association between age and PWMH and DWMH severity based on the Fazekas grading scale (R = 0.3, p < 0.001). After adjusting for age, CMC (1 point for each of the 7 conditions) is associated with PWMH and DWMH severity based on the Fazekas grading scale (R = 0.13, p < 0.001 and R = 0.16, p < 0.001, respectively). Figure 2 also shows a negative association after adjustment for age between decreased cognitive functioning, represented by the global z-score, and increased PWMH and DWMH burden as per the Fazekas rating scale (R = −0.11, p < 0.001 and R = −0.14, p < 0.001, respectively). Furthermore, the plots for PWMH burden showed similar levels of associations with age, CMC, and cognition and visible drop offs in age-associated increase in scores from 1 to 2.

### Association of amyloid-PET, tau-PET, and total intracranial WMH percentage with PWMH and DWMH burden

**Figure 3:**
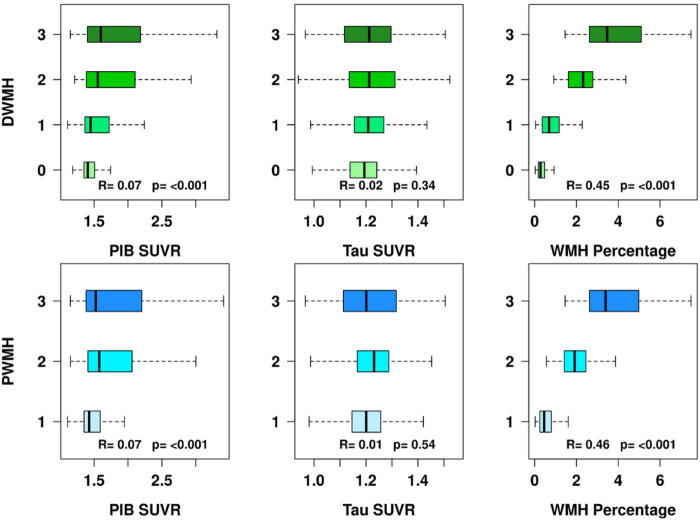
Box plots of amyloid SUVR (standardized uptake value ratio) representing amyloid burden, tau SUVR representing tau burden, and percentage of total brain WMH (white matter hyperintensity) comprised of DWMH and PWMH burden as a function of DWMH and PWMH Fazekas rating scales. The Kendall tau correlations and p-values are listed.

Boxplot in Fig. 3 illustrates the partial Kendall tau correlation of WMH subtypes with amyloid and tau. There is a small but significant positive correlation between increased amyloid burden and both PWMH and DWMH burden (R = 0.07, p < 0.001) after adjusting for age. However, there are no statistically significant associations between increased tau burden, represented by tau SUVR, with both PWMH and DWMH severity (p > 0.3). As an expected measure of internal validity, an increase in PWMH and DWMH burden on the Fazekas scale correlates with increased WMH as a percentage of total intracranial volume Fig. 3.

### Combinability of the 2D GE FLAIR and 3D Siemens FLAIR images

We found that PWMH had a weighted Kappa of 0.7114 (ICC = 0.815) and DWMH had a weighted Kappa of 0.6258 (ICC = 0.741) when comparing the Siemens and GE assessments. Overall, there was an excellent agreement with the same Fazekas score in 62 (87%) participants for PWMH and 54 (76%) participants for DWMH. Hence, we combined data from GE and Siemens scanners for the final analyses and included only the Siemens scans for the cross-over participants. We also conducted sensitivity analyses to confirm our overall findings on GE and Siemens scans separately.

**Figure 4:**
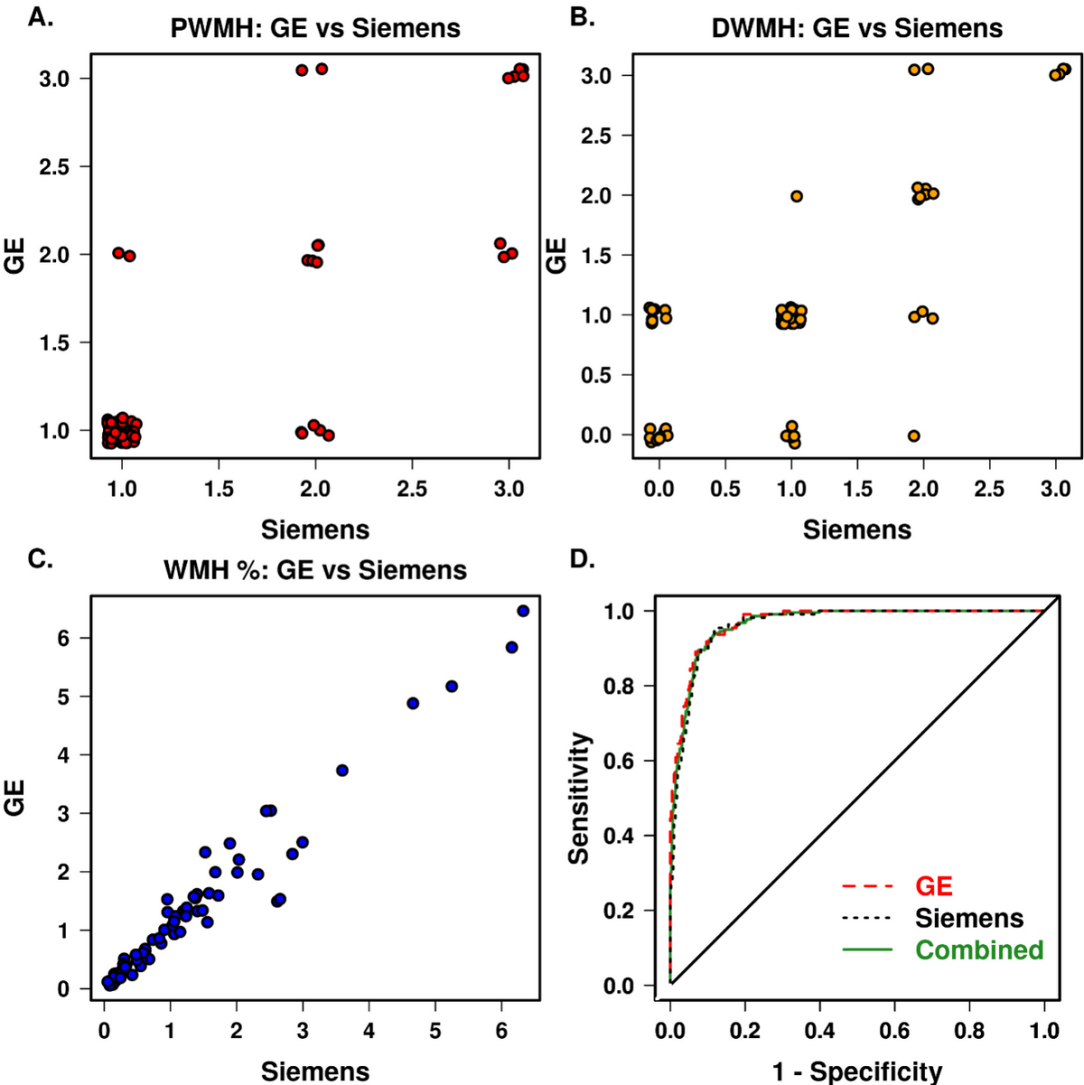
Comparison between information provided by GE and Siemens FLAIR images **(A)** For PWMH, **(B)** For DWMH, **(C)** automated WMH in the cross-over participants, and **(D)** AUROC for Fazekas ≥2 classification using automated WMH in the full sample.

Figure 4A and Fig. 4B show the distribution of ratings of DWMH and PWMH on GE vs. Siemens. In addition, we evaluated the similarity of information given from 2D FLAIR and 3D FLAIR images based on automated WMH detection on the cross-over participants data and found high Pearson correlation r = 0.98 (Fig. 4C).

In the full sample, we further evaluated how well the automated measurements separated Fazekas total score greater than or equal to 2 which is an indicator of VCID. The receiver operating characteristic (ROC) curve analysis shown in Fig. 4D displays the sensitivity and specificity for both 2D GE and 3D Siemens scanners when measuring the percent WMH of total intracranial volume used to discriminate between normal and abnormal Fazekas. In Fig. 4D, the AUROC for (1) GE was 0.970 (p < 0.001), (2) Siemens was 0.963 (p < 0.001), and (3) combined was 0.966 (p < 0.001), indicating minimal to no difference among the three in discriminating VCID normal vs. abnormal groups which we think is justifiable for combining data across MR manufacturers. The optimal cutoff was 1.15% combined, 1.30% for 2D FLAIR images, and 1.15% for 3D FLAIR images.

## DISCUSSION

We assessed the spatial patterns of WMH using Fazekas rating scale and then investigated their associations with underlying etiologies in a population-based cohort. The main conclusions are: (1) information provided by DWMH and PWMH are highly correlated and similar across the associations we tested; (2) both PWMH ≥ 2 and DWMH ≥ 2 are often seen in participants greater than 80 years of age, greater than 2 cardiovascular metabolic conditions, and global z-score < 0; (3) we found a weak positive correlation of PWMH and DWMH burden with amyloid-PET measures, but not with tau-PET measures, and these associations did not differ between PWMH and DWMH; (4) 2D GE vs. 3D Siemens FLAIR did not yield substantial differences in WMH assessments of both visual and automated measurements, supporting the decision to pool data; (5) the automated assessments were able to predict abnormal Fazekas scale on both 2D and 3D FLAIR images with similar AUROC.

### Association of Fazekas measures with age and vascular risk

It is well-established that age plays a pivotal role in the prevalence and severity of WMH, with strong evidence for increased WMH presence on MRI with increased age. In alignment with previous literature on WMH [26, 27], we found significant association between age and severity of PWMH and DWMH graded via the Fazekas scale. Furthermore, studies such as the retrospective investigation conducted by Zhuang et al., provided further clarity on the independent role of age as a risk factor for prevalence and severity of WMH, even after controlling for sex, education, hyperlipidemia, and hyperhomocysteinemia [28]. WMH were previously known to be a normal consequence of aging with limited clinical importance [29]. However, in recent years, multiple studies have established the connection between deteriorating vascular health and increased WMH. Notably, WMH tend to develop in areas characterized by low cerebral perfusion [29, 30] and exhibit heightened prevalence and severity in the presence of conditions like hypertension [31–33], diabetes [34], and recurrent risk of stroke [35].

Our study shows significant correlations for both PWMH and DWMH with higher age and with CMC burden after adjusting for age, suggesting a pathophysiologic involvement of diffusely increased WMH, rather than localization of WMH changes to periventricular or deep regions as a response to worsening vascular health and age. In addition to age, hypertension, especially elevated systolic blood pressure, is a well-known factor linked with WMH presence, especially in adults > 65 years [36]. This is strengthened by the fact that WMH are formed from endothelial activation, inflammation, and ischemic damage, especially increased central arterial stiffness and pressure and increased cerebral blood flow pulsatility [37, 38], all of which are worsened by hypertension [36]. The duration of hypertension also has an incremental impact on WMH, as suggested by de Leeuw et al. 2002 who demonstrated that periventricular and subcortical WMH are associated with duration of hypertension [5]. While controlling blood pressure with antihypertensives is widely known to reduce cardiovascular morbidity and mortality risk among endless health benefits, their role in preventing WMH is not unanimous. Some studies demonstrate that successful control of hypertension (lifestyle modifications, antihypertensives) reduces the risk of dementia [36] and white matter lesions [39]. However, results from the Cardiovascular Determinants of Dementia (CASCADE) study, a multicenter European collaborative study, revealed that sudden hypoperfusion from aggressive blood pressure management in chronic hypertension cases increases further risk of WMH [32]. This is attributed to vascular remodeling and hyalinosis over time that increases flow requirements to maintain adequate perfusion, which can be disrupted by sudden control. Overall, our study reinforces the established connections between Fazekas WMH grading, age, and CMC, shedding light on the intricate interplay between these factors in the context of white matter integrity in the brain.

### Association of Fazekas WMH measures with AD imaging biomarkers

WMH are known to be more severe and prevalent in individuals with AD compared to healthy controls of the same age [40, 41]. Currently, two theories for the involvement of WMH in AD exist: (1) *additive* theory, where WMH is primarily due to cerebrovascular disease and it lowers the threshold for AD diagnosis independent of AD pathology, and (2) *interactive* theory, where WMHs interact with amyloid/tau to potentiate their effects on cognitive impairment [19]. There is also a distinct spatial pattern for WMH in AD individuals, with a greater involvement of posterior parieto-occipital and periventricular areas, compared to healthy controls of the same age [42–44]. Potential hypothesized mechanisms underlying formation of WMH in AD include Wallerian degeneration secondary to tau neurofibrillary tangles [45], overlap of amyloidosis from CAA with AD [19], and neuroinflammation, although this has mostly been observed in SVD [46].

A previous study by our group as well as others have established a link between WMH and amyloid burden, localized to regions with lobar cerebral microbleeds, indicating the involvement of CAA in increased WMH [18, 36]. The current study further investigates this association by investigating differential involvement of PWMH and DWMH, quantified according to standardized Fazekas scale ratings, with amyloid and tau burden with a large number of participants. Although the association was minimal, our study identified a correlation between increased DWMH and PWMH burden with amyloid burden. Furthermore, neither PWMH nor DWMH had a significant association with tau burden, which contrasts findings in McAleese et al., where they demonstrated an independent influence of cortical tau burden on the severity of WMH [20]. This disparity in results is likely due to differences in the characteristics of study populations. Our cohort primarily comprises of cognitively unimpaired individuals with lower tau burden. However, our population characteristics are reflective of the general population that enabled us to investigate the influence of AD pathology influences on these two common patterns of WMH.

### Association of Fazekas WMH measures with cognition

Consistent with prior literature that reported association between worsening cognitive impairment and increased WMH burden [48], we also found significant negative relationship between periventricular and deep WMH with global cognition. Poor vascular health, especially cerebrovascular, significantly contributes to worsening cognitive impairment and rates of dementia [49]. Furthermore, the impact of WMH observed on cognitive function has been evaluated globally [49] as well as differentially on specific cognitive domains, executive dysfunction [49–56] and inconsistent memory impairment [49, 51, 54, 57–59].

A more recent study has made efforts to create comprehensive neural mapping of structures that directly correlate WMH to domain-specific cognitive task impairments. The anterior thalamic radiations, forceps major, and left inferior fronto-occipital fasciculus are major drivers of decline in tasks related to executive function, information processing, language, and verbal memory [60]. While Coenen et al. 2023 found that cognitive impairment was independent of total WMH volume loss, another recent study regards WMH volume change as a more dynamic process that can help predict cognitive dysfunction in individuals with minor strokes, which is a common comorbidity in those with WMH [61]. Of note, WMH shape including confluence, rather than volume, may also play a role in increased executive dysfunction and memory impairment, as reported by Zwartbol et al. 2022 [62].

### Low Distinction between PWMH and DWMH

In the present study, global cognition was associated with PWMH and DWMH burden, demonstrating an equal level of significance for both, indicating the collective impact of diffuse WMH changes across the white matter networks in the brain on cognitive decline, rather than localization to periventricular or subcortical regions. Our findings suggest an absence of differential involvement of DWMH or PWMH in VCID, given both scorings’ significant association with CMC and cognitive impairment. This aligns with established findings of global cognitive impairment in multiple domains rising from equal dysfunction and damage to both WMH from deeper axonal tract and periventricular regions. In contrast, a recent meta-analysis by Botz et al. [10] reported a distinctive spatial correlation between WMH and cognition using ROI, visual rating, and voxel-wise approaches, suggesting the spatial distinction as an early marker of cognitive impairment in younger individuals. The observed contrasting findings might be due to the difference in the studied population including cognitively normal, participants with MCI or AD dementia, while we included a population-based sample in which only 13% were cognitively impaired.

### Strengths and Limitations

The main strength of this study is its extensive analysis of the relationship of both PWMH and DWMH and cognition, cerebrovascular health, and amyloid and tau burden. While these correlations have previously been investigated and assessed, this study uniquely divides WMH into its subtypes based on localization in the brain and attaching a standardized, quantitative scale to appropriately classify the extent and severity of WMH presence. The characteristics of the population-based sample in this study strengthen the generalizability of the results of this study. In the interest of maximizing our sample size and thus the power of this study, we tested whether we could combine data from our GE 2D FLAIR and Siemens 3D FLAIR subsamples. The comparative sensitivity and specificity of each were determined and compared, both individually and combined, yielding satisfactory results. However, it should be noted that our in-house WMH measurement algorithm contains steps to internally adjust for manufacturer and 2D vs 3D FLAIR in its assessments, developed based on previous analyses of our head-to-head crossover study. Readers should not incorrectly conclude that these automated measurements would be directly combinable or comparable if they came from other WMH measurement software and/or without steps for scanner harmonization.

Our study has certain limitations. First, the cross-sectional design of the study restricts our ability to establish cause-effect relationship between age, CMC, AD biomarkers and the spatial distribution of WMH. Second, risk factors such as BP, HbA1c levels, smoking and drinking were not considered. Third, although the lobar regional WMH measures may be more relevant in the context of AD pathology, we focused solely on the contribution of periventricular and deep WMH to global AD biomarkers, although the Fazekas scale is a widespread, established measure using the two aforementioned divisions. Lastly, the relatively small number of individuals with a Fazekas grade of 3 may impact the generalizability of our findings. Future longitudinal studies will target how regional distribution of WMHs influences the pathophysiological cascade of AD and dementia, with a specific focus on discerning the differential involvement and association of cognitive domains such as attention and memory that with WMH burden.

## CONCLUSION

In conclusion, our study contributes to the fund of knowledge currently present regarding the utility of the Fazekas grading scale in not only conventionally assessing WMH severity, but also correlating different WMH spatial distributions with AD neuropathological markers. It demonstrates a strong association between amyloidosis with both PWMH and DWMH, but minimal to no statistical differences between degree of correlations between PWMH and DWMH. These findings, along with ones regarding inter-scanner combinability, will guide the development of predictive models for those at risk for either early stages or future development of AD, ultimately informing on how they can be implemented into clinical care for more meaningful outcomes in disease.

## Figures and Tables

**Table 1:** Characteristics of sample with mean (standard deviation) listed for continuous variables and count (%) for categorical variables.

	All n = 1144	GE (2D FLAIR) n = 524	Siemens (3D FLAIR) n = 620	*p*-value
Age, years	74.0 (8.8)	74.7 (8.6)	73.5 (9.0)	0.026
Males, no. (%)	604 (53%)	280 (53%)	324 (52%)	0.73
*APOe4*, no. (%)	306 (29%)	146 (28%)	160 (30%)	0.56
Education, years	14.9 (2.6)	14.8 (2.6)	15.1 (2.5)	0.066
MMSE	28.3 (1.8)	28.3 (1.6)	28.2 (2.0)	0.15
Global cognitive z-score	0.10 (1.20)	0.13 (1.06)	0.08 (1.31)	0.042
Amyloid, SUVR	1.62 (0.44)	1.62 (0.42)	1.63 (0.45)	0.14
Amyloid Positive, no. (%)	483 (42%)	229 (44%)	254 (41 %)	0.97
Tau, SUVR	1.22 (0.13)	1.21 (0.12)	1.22 (0.14)	0.10
Tau Positive, no. (%)	340 (30%)	147 (28%)	193 (31%)	0.067
CMC	2.1 (1.3)	2.1 (1.3)	2.0 (1.3)	0.50
Abnormal CMC, no. (%)	682 (64%)	347 (66%)	335 (62%)	0.48
Abnormal Fazekas, no. (%)	222 (19%)	110 (21 %)	112 (18%)	0.50
PWMH	1.2 (0.5)	1.3 (0.6)	1.2 (0.5)	0.47
1, no. (%)	922 (81%)	414 (79%)	508 (82%)	
2, no. (%)	165 (14%)	79 (15%)	86 (14%)	
3, no. (%)	57 (5%)	31 (6%)	26 (4%)	
DWMH	0.9 (0.7)	0.9 (0.8)	0.8 (0.7)	0.082
0, no. (%)	355 (31%)	148 (28%)	207 (33%)	
1, no. (%)	651 (57%)	304 (58%)	347 (56%)	
2, no. (%)	87 (8%)	42 (8%)	45 (7%)	
3, no. (%)	51 (4%)	30 (6%)	21 (3%)	
WMH percentage	0.95 (1.02)	0.98 (1.12)	0.92 (0.93)	0.004
Cognitively Impaired, no. (%)	150 (13%)	49 (9%)	101 (16%)	<0.001

p-values come from an ANCOVA for continuous variables and a logistic regression for categorical variables with an age adjustment. **CMC** = cardiovascular and metabolic conditions; **MMSE** = mini mental status examination; SUVR = standardized uptake value ratio; **VCID** = vascular contributions to cognitive impairment and dementia

## Data Availability

The datasets used and analyzed for this study are made available by the corresponding author upon reasonable request due to study participant privacy.

## Abbreviations

AD: Alzheimer’s disease
ANCOVA: Analysis of covariance
AUROC: Area under the receiver operating characteristic curve
CASCADE: Cardiovascular Determinants of Dementia
CMC: Cardiometabolic condition
DWMH: Deep white matter hyperintensities
FLAIR: Fluid-attenuated inversion recovery
ICC: Intraclass correlations
IRB: Institutional Review Board
MCSA: Mayo Clinic Study of Aging
MRI: Magnetic resonance imaging
PET: Positron emission tomography
PWMH: Periventricular white matter hyperintensities
ROI: Region of interest
SD: Standard deviation
SVD: Small vessel disease
SUVR: Standardized uptake value ratio
VCID: Vascular contributions to cognitive impairment and dementia
WMH: White matter hyperintensities
WMS-R: Wechsler Memory Scale–Revised
WAIS-R: Wechsler Adult Intelligence Scale–Revised
